# (20*R*,24*R*,25*S*)-3α,7α,12α,27-Tetra­acet­oxy-24,26-ep­oxy-5β-cholestane

**DOI:** 10.1107/S160053680901188X

**Published:** 2009-04-10

**Authors:** Kamal Aziz Ketuly, A. Hamid A. Hadi, Seik Weng Ng

**Affiliations:** a Department of Chemistry, University of Malaya, 50603 Kuala Lumpur, Malaysia

## Abstract

In the title anhydro­scymnol tetra­acetate, C_35_H_54_O_9_, the fused chair conformation of the cyclo­hexane *A*/*B* ring junction is *cis* with a 5β-*H* configuration. The compound has a trimethyl­ene oxide ring at position 24,26 and four acetate groups at the 3α,7α,12α,27 positions.

## Related literature

For the synthesis from shark bile sterol sodium scymnol sulfate, see: Cross (1961 ▶). For the assignment of the absolute configuration of the carbon at the 20-position in (20*S*)-6β-meth­oxy-20-(*p*-toluene­sulfoxymeth­yl)-3α,5-cyclo-5α-pregnane see: Ketuly *et al.* (1997 ▶). For the crystal structure of the unacetyl­ated anhydro­scymnol, see: Ishida *et al.* (1991 ▶, 1994 ▶); other studies have not mentioned the configuration at C20.
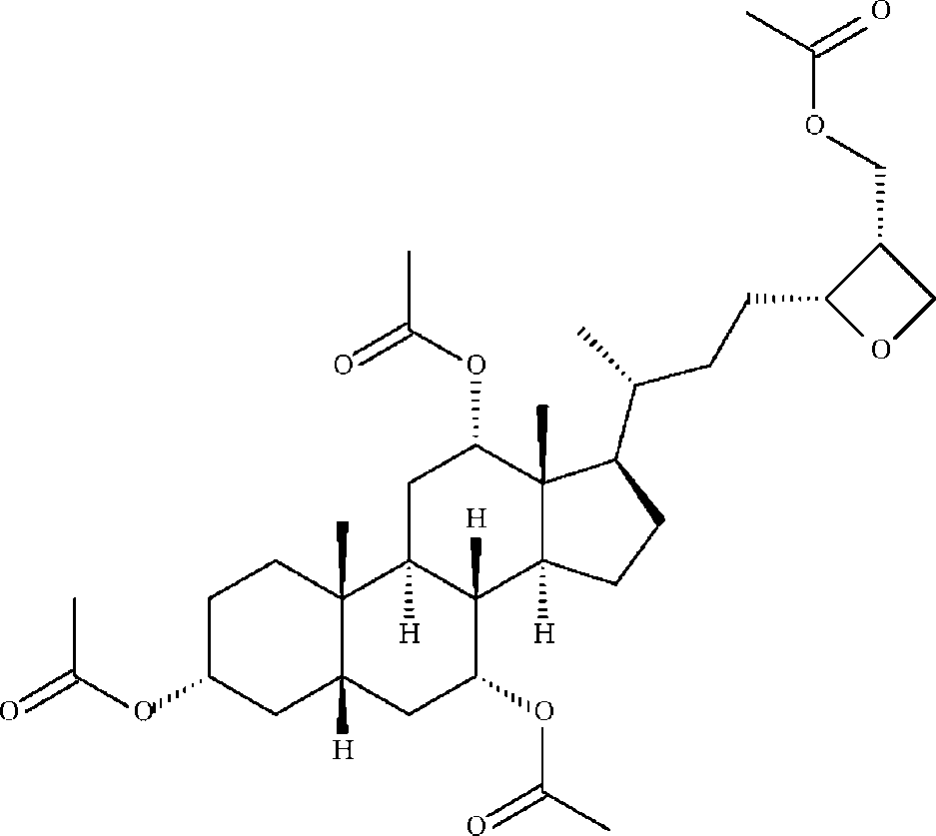


## Experimental

### 

#### Crystal data

C_35_H_54_O_9_
*M* *_r_* = 618.78
Orthorhombic, 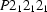

*a* = 10.9474 (2) Å
*b* = 14.7638 (2) Å
*c* = 20.4477 (3) Å
*V* = 3304.86 (9) Å^3^
*Z* = 4
Mo *K*α radiation
μ = 0.09 mm^−1^
*T* = 100 K
0.35 × 0.30 × 0.25 mm

#### Data collection

Bruker SMART APEX diffractometer
Absorption correction: none
28923 measured reflections
4552 independent reflections
4212 reflections with *I* > 2σ(*I*)
*R*_int_ = 0.031

#### Refinement

*R*[*F* ^2^ > 2σ(*F* ^2^)] = 0.032
*wR*(*F* ^2^) = 0.085
*S* = 1.01
4552 reflections
404 parameters
H-atom parameters constrained
Δρ_max_ = 0.29 e Å^−3^
Δρ_min_ = −0.16 e Å^−3^

### 

Data collection: *APEX2* (Bruker, 2008 ▶); cell refinement: *SAINT* (Bruker, 2008); data reduction: *SAINT*; program(s) used to solve structure: *SHELXS97* (Sheldrick, 2008 ▶); program(s) used to refine structure: *SHELXL97* (Sheldrick, 2008 ▶); molecular graphics: *X-SEED* (Barbour, 2001 ▶); software used to prepare material for publication: *publCIF* (Westrip, 2009 ▶).

## Supplementary Material

Crystal structure: contains datablocks global, I. DOI: 10.1107/S160053680901188X/tk2410sup1.cif

Structure factors: contains datablocks I. DOI: 10.1107/S160053680901188X/tk2410Isup2.hkl

Additional supplementary materials:  crystallographic information; 3D view; checkCIF report

## Figures and Tables

**Fig. 1.**
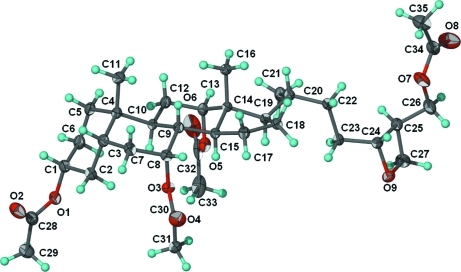
Thermal ellipsoid plot (Barbour, 2001) of C35H54O9 at the 70% probability level. Hydrogen atoms are drawn as spheres of arbitrary radius.

## supplementary crystallographic information

### Experimental

Anhydroscymnol (1 mmol) was reacted with acetic anhydride (1.3 mmol) in dry pyridine at 343 K for 2 hours. Water was added to the mixture, and the product extracted with ethyl acetate. The solvent was removed and the product was fractionated over a silica gel column and eluted with hexane:ethyl acetate (3:1 v/v). The semicrystalline fraction was recrystallized with hexane:ethyl acetate; m.p. 423–425 K [Lit. 420–423 K (Cross, 1961)].

### Refinement

Hydrogen atoms were placed at calculated positions (C–H 0.95–0.99 Å) and were treated as riding on their parent carbon atoms, with *U*(H) set to 1.2–1.5 times *U*_eq_(*C*).

The absolute configuration of the carbon atom at position 20 is based on the absolute configuration of (20*S*)-6β-methoxy-20-(*p*-toluenesulfoxymethyl)-3α,5-cyclo-5α-pregnane (Ketuly *et al.*, 1997).

### Crystal data

**Table:**

C_35_H_54_O_9_	*F*(000) = 1344
*M**_r_* = 618.78	*D*_x_ = 1.244 Mg m^−^^3^
Orthorhombic, *P*2_1_2_1_2_1_	Mo *K*α radiation, λ = 0.71073 Å
Hall symbol: P 2ac 2ab	Cell parameters from 9970 reflections
*a* = 10.9474 (2) Å	θ = 2.5–28.1°
*b* = 14.7638 (2) Å	µ = 0.09 mm^−^^1^
*c* = 20.4477 (3) Å	*T* = 100 K
*V* = 3304.86 (9) Å^3^	Block, colorless
*Z* = 4	0.35 × 0.30 × 0.25 mm

### Data collection

**Table:**

Bruker SMART APEX diffractometer	4212 reflections with *I* > 2σ(*I*)
Radiation source: fine-focus sealed tube	*R*_int_ = 0.031
graphite	θ_max_ = 28.3°, θ_min_ = 1.7°
ω scans	*h* = −14→14
28923 measured reflections	*k* = −19→19
4552 independent reflections	*l* = −27→27

### Refinement

**Table:**

Refinement on *F*^2^	Primary atom site location: structure-invariant direct methods
Least-squares matrix: full	Secondary atom site location: difference Fourier map
*R*[*F*^2^ > 2σ(*F*^2^)] = 0.032	Hydrogen site location: inferred from neighbouring sites
*wR*(*F*^2^) = 0.085	H-atom parameters constrained
*S* = 1.00	*w* = 1/[σ^2^(*F*_o_^2^) + (0.0562*P*)^2^ + 0.4501*P*] where *P* = (*F*_o_^2^ + 2*F*_c_^2^)/3
4552 reflections	(Δ/σ)_max_ = 0.001
404 parameters	Δρ_max_ = 0.29 e Å^−^^3^
0 restraints	Δρ_min_ = −0.16 e Å^−^^3^

### Special details

**Table:**

Geometry. All esds (except the esd in the dihedral angle between two l.s. planes) are estimated using the full covariance matrix. The cell esds are taken into account individually in the estimation of esds in distances, angles and torsion angles; correlations between esds in cell parameters are only used when they are defined by crystal symmetry. An approximate (isotropic) treatment of cell esds is used for estimating esds involving l.s. planes.

### Fractional atomic coordinates and isotropic or equivalent isotropic displacement parameters (Å^2^)

**Table:**

	*x*	*y*	*z*	*U*_iso_*/*U*_eq_	
O1	0.68083 (10)	−0.09988 (7)	0.82696 (6)	0.0191 (2)	
O2	0.53071 (12)	−0.18393 (9)	0.87116 (7)	0.0294 (3)	
O3	0.70747 (10)	0.17227 (7)	0.96918 (5)	0.0168 (2)	
O4	0.65553 (13)	0.15801 (9)	1.07531 (6)	0.0288 (3)	
O5	0.91968 (10)	0.28801 (8)	0.82837 (6)	0.0192 (2)	
O6	1.00233 (15)	0.27411 (10)	0.72826 (7)	0.0399 (4)	
O7	1.13334 (12)	0.85069 (8)	0.92430 (7)	0.0295 (3)	
O8	1.19343 (14)	0.96010 (11)	0.85535 (8)	0.0431 (4)	
O9	1.29555 (10)	0.62411 (8)	0.99315 (6)	0.0209 (2)	
C1	0.59639 (14)	−0.02458 (10)	0.81801 (8)	0.0179 (3)	
H1	0.5146	−0.0482	0.8044	0.021*	
C2	0.58408 (15)	0.02767 (10)	0.88158 (8)	0.0178 (3)	
H2A	0.6660	0.0473	0.8964	0.021*	
H2B	0.5495	−0.0126	0.9156	0.021*	
C3	0.50121 (14)	0.11141 (10)	0.87363 (7)	0.0168 (3)	
H3	0.4178	0.0884	0.8627	0.020*	
C4	0.54085 (14)	0.17409 (10)	0.81621 (8)	0.0165 (3)	
C5	0.56181 (15)	0.11502 (11)	0.75483 (8)	0.0201 (3)	
H5A	0.4818	0.0917	0.7398	0.024*	
H5B	0.5950	0.1539	0.7196	0.024*	
C6	0.64795 (16)	0.03487 (11)	0.76451 (8)	0.0194 (3)	
H6A	0.6552	0.0000	0.7234	0.023*	
H6B	0.7302	0.0567	0.7770	0.023*	
C7	0.48981 (14)	0.16309 (10)	0.93883 (8)	0.0178 (3)	
H7A	0.4144	0.2001	0.9376	0.021*	
H7B	0.4806	0.1184	0.9746	0.021*	
C8	0.59733 (14)	0.22508 (10)	0.95498 (7)	0.0155 (3)	
H8	0.5763	0.2633	0.9938	0.019*	
C9	0.63102 (14)	0.28605 (10)	0.89768 (7)	0.0143 (3)	
H9	0.5580	0.3242	0.8874	0.017*	
C10	0.65823 (14)	0.22709 (10)	0.83682 (7)	0.0136 (3)	
H10	0.7191	0.1806	0.8510	0.016*	
C11	0.43540 (15)	0.23919 (12)	0.79937 (9)	0.0223 (3)	
H11A	0.3674	0.2048	0.7806	0.033*	
H11B	0.4638	0.2842	0.7676	0.033*	
H11C	0.4080	0.2700	0.8392	0.033*	
C12	0.71774 (15)	0.27970 (11)	0.78018 (7)	0.0172 (3)	
H12A	0.7507	0.2358	0.7481	0.021*	
H12B	0.6540	0.3158	0.7578	0.021*	
C13	0.82089 (14)	0.34333 (10)	0.80156 (7)	0.0161 (3)	
H13	0.8512	0.3788	0.7632	0.019*	
C14	0.77911 (13)	0.40776 (10)	0.85570 (7)	0.0136 (3)	
C15	0.73613 (13)	0.34986 (10)	0.91417 (7)	0.0134 (3)	
H15	0.8068	0.3113	0.9277	0.016*	
C16	0.67683 (15)	0.46758 (11)	0.82692 (8)	0.0190 (3)	
H16A	0.6034	0.4308	0.8200	0.029*	
H16B	0.7038	0.4930	0.7851	0.029*	
H16C	0.6583	0.5169	0.8574	0.029*	
C17	0.71512 (15)	0.41932 (10)	0.96898 (8)	0.0172 (3)	
H17A	0.7308	0.3921	1.0124	0.021*	
H17B	0.6304	0.4428	0.9679	0.021*	
C18	0.80838 (14)	0.49564 (10)	0.95403 (7)	0.0163 (3)	
H18A	0.8669	0.5022	0.9906	0.020*	
H18B	0.7656	0.5541	0.9478	0.020*	
C19	0.87656 (14)	0.46804 (10)	0.89041 (7)	0.0143 (3)	
H19	0.9465	0.4282	0.9031	0.017*	
C20	0.92883 (14)	0.54939 (10)	0.85230 (8)	0.0160 (3)	
H20	0.8579	0.5826	0.8330	0.019*	
C21	1.01046 (16)	0.51931 (11)	0.79530 (8)	0.0207 (3)	
H21A	1.0552	0.5717	0.7782	0.031*	
H21B	0.9597	0.4934	0.7606	0.031*	
H21C	1.0687	0.4736	0.8107	0.031*	
C22	0.99790 (15)	0.61701 (10)	0.89648 (8)	0.0172 (3)	
H22A	1.0201	0.6706	0.8699	0.021*	
H22B	0.9415	0.6378	0.9312	0.021*	
C23	1.11407 (15)	0.58050 (10)	0.92896 (8)	0.0179 (3)	
H23A	1.0920	0.5345	0.9622	0.021*	
H23B	1.1663	0.5510	0.8957	0.021*	
C24	1.18393 (14)	0.65683 (10)	0.96117 (8)	0.0168 (3)	
H24	1.1314	0.6928	0.9916	0.020*	
C25	1.25942 (15)	0.71781 (11)	0.91536 (8)	0.0190 (3)	
H25	1.2466	0.7044	0.8679	0.023*	
C26	1.25669 (16)	0.81756 (11)	0.93112 (11)	0.0277 (4)	
H26A	1.2856	0.8276	0.9764	0.033*	
H26B	1.3115	0.8508	0.9010	0.033*	
C27	1.37105 (16)	0.66966 (12)	0.94467 (9)	0.0243 (4)	
H27A	1.4113	0.6275	0.9138	0.029*	
H27B	1.4314	0.7118	0.9641	0.029*	
C28	0.63620 (16)	−0.17487 (11)	0.85596 (8)	0.0207 (3)	
C29	0.73426 (17)	−0.24348 (12)	0.86679 (9)	0.0252 (4)	
H29A	0.6974	−0.3036	0.8718	0.038*	
H29B	0.7800	−0.2281	0.9064	0.038*	
H29C	0.7898	−0.2439	0.8292	0.038*	
C30	0.72559 (15)	0.14408 (10)	1.03085 (8)	0.0185 (3)	
C31	0.84298 (16)	0.09206 (11)	1.03599 (9)	0.0231 (3)	
H31A	0.8674	0.0879	1.0820	0.035*	
H31B	0.9068	0.1233	1.0111	0.035*	
H31C	0.8313	0.0310	1.0182	0.035*	
C32	1.00340 (17)	0.25635 (12)	0.78586 (10)	0.0270 (4)	
C33	1.09408 (17)	0.19690 (14)	0.81905 (12)	0.0389 (5)	
H33A	1.1269	0.1532	0.7876	0.058*	
H33B	1.0542	0.1644	0.8550	0.058*	
H33C	1.1608	0.2340	0.8365	0.058*	
C34	1.11389 (17)	0.92218 (11)	0.88489 (8)	0.0229 (3)	
C35	0.98074 (18)	0.94542 (13)	0.88180 (10)	0.0304 (4)	
H35A	0.9713	1.0108	0.8753	0.046*	
H35B	0.9411	0.9276	0.9228	0.046*	
H35C	0.9427	0.9130	0.8453	0.046*	

### Atomic displacement parameters (Å^2^)

**Table:**

	*U*^11^	*U*^22^	*U*^33^	*U*^12^	*U*^13^	*U*^23^
O1	0.0182 (5)	0.0162 (5)	0.0229 (5)	0.0002 (4)	0.0016 (4)	−0.0014 (4)
O2	0.0229 (6)	0.0231 (6)	0.0421 (8)	0.0000 (5)	0.0072 (6)	0.0067 (6)
O3	0.0157 (5)	0.0189 (5)	0.0157 (5)	0.0019 (4)	0.0015 (4)	0.0019 (4)
O4	0.0374 (7)	0.0318 (7)	0.0172 (6)	0.0029 (6)	0.0043 (5)	0.0002 (5)
O5	0.0146 (5)	0.0204 (5)	0.0225 (6)	−0.0001 (4)	0.0033 (4)	−0.0054 (5)
O6	0.0486 (9)	0.0355 (7)	0.0356 (8)	−0.0047 (7)	0.0239 (7)	−0.0119 (6)
O7	0.0256 (6)	0.0192 (6)	0.0438 (8)	0.0011 (5)	0.0051 (6)	0.0090 (6)
O8	0.0308 (7)	0.0471 (9)	0.0515 (9)	−0.0036 (7)	0.0047 (7)	0.0246 (8)
O9	0.0181 (5)	0.0223 (6)	0.0224 (6)	−0.0028 (5)	−0.0049 (5)	0.0037 (5)
C1	0.0170 (7)	0.0155 (7)	0.0212 (8)	−0.0003 (6)	−0.0003 (6)	−0.0007 (6)
C2	0.0194 (7)	0.0147 (7)	0.0194 (7)	−0.0011 (6)	0.0019 (6)	0.0005 (6)
C3	0.0133 (6)	0.0171 (7)	0.0201 (7)	−0.0026 (6)	0.0006 (6)	0.0001 (6)
C4	0.0137 (7)	0.0162 (7)	0.0195 (7)	−0.0028 (6)	−0.0028 (6)	0.0007 (6)
C5	0.0227 (8)	0.0196 (7)	0.0179 (7)	−0.0056 (7)	−0.0042 (6)	−0.0007 (6)
C6	0.0225 (8)	0.0197 (7)	0.0159 (7)	−0.0044 (6)	0.0019 (6)	−0.0025 (6)
C7	0.0142 (7)	0.0170 (7)	0.0223 (7)	−0.0005 (6)	0.0051 (6)	−0.0004 (6)
C8	0.0143 (7)	0.0158 (7)	0.0164 (7)	0.0018 (6)	0.0027 (5)	−0.0002 (6)
C9	0.0130 (6)	0.0142 (6)	0.0157 (7)	−0.0011 (5)	0.0009 (5)	0.0004 (5)
C10	0.0130 (6)	0.0135 (6)	0.0144 (7)	−0.0017 (5)	−0.0010 (5)	0.0000 (5)
C11	0.0172 (7)	0.0217 (8)	0.0280 (9)	−0.0014 (6)	−0.0069 (7)	0.0027 (7)
C12	0.0200 (7)	0.0180 (7)	0.0136 (7)	−0.0048 (6)	−0.0008 (6)	0.0005 (6)
C13	0.0173 (7)	0.0162 (7)	0.0147 (7)	−0.0039 (6)	0.0012 (6)	−0.0011 (6)
C14	0.0132 (6)	0.0129 (6)	0.0148 (7)	−0.0008 (6)	−0.0019 (6)	0.0005 (5)
C15	0.0124 (6)	0.0136 (6)	0.0141 (7)	−0.0005 (5)	0.0002 (5)	−0.0001 (5)
C16	0.0168 (7)	0.0167 (7)	0.0236 (8)	−0.0008 (6)	−0.0058 (6)	0.0027 (6)
C17	0.0172 (7)	0.0171 (7)	0.0174 (7)	0.0000 (6)	0.0013 (6)	−0.0025 (6)
C18	0.0181 (7)	0.0132 (6)	0.0174 (7)	0.0009 (6)	−0.0009 (6)	−0.0011 (5)
C19	0.0136 (6)	0.0142 (6)	0.0150 (7)	0.0003 (5)	−0.0015 (5)	−0.0001 (5)
C20	0.0158 (7)	0.0139 (6)	0.0184 (7)	−0.0009 (6)	−0.0015 (6)	0.0010 (6)
C21	0.0236 (8)	0.0194 (7)	0.0190 (7)	−0.0059 (7)	0.0027 (6)	−0.0006 (6)
C22	0.0177 (7)	0.0134 (6)	0.0206 (7)	0.0005 (6)	−0.0026 (6)	−0.0003 (6)
C23	0.0190 (7)	0.0134 (7)	0.0212 (8)	−0.0024 (6)	−0.0027 (6)	0.0005 (6)
C24	0.0170 (7)	0.0167 (7)	0.0168 (7)	−0.0012 (6)	−0.0013 (6)	0.0007 (6)
C25	0.0198 (7)	0.0178 (7)	0.0194 (7)	−0.0028 (6)	−0.0002 (6)	0.0003 (6)
C26	0.0226 (8)	0.0176 (8)	0.0429 (10)	−0.0041 (7)	−0.0020 (8)	0.0021 (7)
C27	0.0194 (8)	0.0236 (8)	0.0299 (9)	0.0000 (7)	0.0009 (7)	0.0048 (7)
C28	0.0250 (8)	0.0173 (7)	0.0196 (8)	0.0003 (6)	0.0015 (6)	−0.0029 (6)
C29	0.0260 (8)	0.0217 (8)	0.0279 (9)	0.0047 (7)	0.0022 (7)	0.0001 (7)
C30	0.0229 (7)	0.0158 (7)	0.0167 (7)	−0.0053 (6)	−0.0031 (6)	−0.0019 (6)
C31	0.0224 (8)	0.0211 (8)	0.0258 (8)	−0.0013 (7)	−0.0075 (7)	0.0020 (7)
C32	0.0204 (8)	0.0226 (8)	0.0378 (10)	−0.0089 (7)	0.0113 (8)	−0.0168 (7)
C33	0.0193 (8)	0.0362 (10)	0.0612 (14)	0.0036 (8)	0.0024 (9)	−0.0261 (10)
C34	0.0307 (9)	0.0185 (7)	0.0196 (8)	−0.0006 (7)	0.0027 (7)	−0.0002 (6)
C35	0.0300 (9)	0.0277 (9)	0.0335 (10)	0.0056 (8)	0.0092 (8)	0.0070 (8)

### Geometric parameters (Å, °)

**Table:**

O1—C28	1.348 (2)	C14—C19	1.560 (2)
O1—C1	1.4574 (19)	C15—C17	1.536 (2)
O2—C28	1.203 (2)	C15—H15	1.0000
O3—C30	1.3427 (19)	C16—H16A	0.9800
O3—C8	1.4650 (18)	C16—H16B	0.9800
O4—C30	1.207 (2)	C16—H16C	0.9800
O5—C32	1.347 (2)	C17—C18	1.551 (2)
O5—C13	1.4619 (19)	C17—H17A	0.9900
O6—C32	1.207 (3)	C17—H17B	0.9900
O7—C34	1.345 (2)	C18—C19	1.554 (2)
O7—C26	1.443 (2)	C18—H18A	0.9900
O8—C34	1.199 (2)	C18—H18B	0.9900
O9—C27	1.455 (2)	C19—C20	1.542 (2)
O9—C24	1.4678 (19)	C19—H19	1.0000
C1—C6	1.512 (2)	C20—C21	1.534 (2)
C1—C2	1.518 (2)	C20—C22	1.544 (2)
C1—H1	1.0000	C20—H20	1.0000
C2—C3	1.542 (2)	C21—H21A	0.9800
C2—H2A	0.9900	C21—H21B	0.9800
C2—H2B	0.9900	C21—H21C	0.9800
C3—C7	1.541 (2)	C22—C23	1.533 (2)
C3—C4	1.557 (2)	C22—H22A	0.9900
C3—H3	1.0000	C22—H22B	0.9900
C4—C11	1.541 (2)	C23—C24	1.513 (2)
C4—C5	1.545 (2)	C23—H23A	0.9900
C4—C10	1.562 (2)	C23—H23B	0.9900
C5—C6	1.526 (2)	C24—C25	1.540 (2)
C5—H5A	0.9900	C24—H24	1.0000
C5—H5B	0.9900	C25—C26	1.508 (2)
C6—H6A	0.9900	C25—C27	1.536 (2)
C6—H6B	0.9900	C25—H25	1.0000
C7—C8	1.527 (2)	C26—H26A	0.9900
C7—H7A	0.9900	C26—H26B	0.9900
C7—H7B	0.9900	C27—H27A	0.9900
C8—C9	1.523 (2)	C27—H27B	0.9900
C8—H8	1.0000	C28—C29	1.492 (2)
C9—C15	1.525 (2)	C29—H29A	0.9800
C9—C10	1.548 (2)	C29—H29B	0.9800
C9—H9	1.0000	C29—H29C	0.9800
C10—C12	1.539 (2)	C30—C31	1.501 (2)
C10—H10	1.0000	C31—H31A	0.9800
C11—H11A	0.9800	C31—H31B	0.9800
C11—H11B	0.9800	C31—H31C	0.9800
C11—H11C	0.9800	C32—C33	1.489 (3)
C12—C13	1.533 (2)	C33—H33A	0.9800
C12—H12A	0.9900	C33—H33B	0.9800
C12—H12B	0.9900	C33—H33C	0.9800
C13—C14	1.529 (2)	C34—C35	1.499 (3)
C13—H13	1.0000	C35—H35A	0.9800
C14—C16	1.543 (2)	C35—H35B	0.9800
C14—C15	1.543 (2)	C35—H35C	0.9800
			
C28—O1—C1	116.87 (12)	C15—C17—H17A	110.9
C30—O3—C8	118.21 (12)	C18—C17—H17A	110.9
C32—O5—C13	117.09 (14)	C15—C17—H17B	110.9
C34—O7—C26	118.17 (14)	C18—C17—H17B	110.9
C27—O9—C24	90.99 (11)	H17A—C17—H17B	109.0
O1—C1—C6	107.27 (13)	C17—C18—C19	106.89 (12)
O1—C1—C2	109.66 (13)	C17—C18—H18A	110.3
C6—C1—C2	110.96 (12)	C19—C18—H18A	110.3
O1—C1—H1	109.6	C17—C18—H18B	110.3
C6—C1—H1	109.6	C19—C18—H18B	110.3
C2—C1—H1	109.6	H18A—C18—H18B	108.6
C1—C2—C3	111.69 (13)	C20—C19—C18	113.40 (12)
C1—C2—H2A	109.3	C20—C19—C14	117.92 (12)
C3—C2—H2A	109.3	C18—C19—C14	101.66 (12)
C1—C2—H2B	109.3	C20—C19—H19	107.8
C3—C2—H2B	109.3	C18—C19—H19	107.8
H2A—C2—H2B	107.9	C14—C19—H19	107.8
C7—C3—C2	110.69 (13)	C21—C20—C19	112.00 (12)
C7—C3—C4	112.39 (12)	C21—C20—C22	110.27 (13)
C2—C3—C4	113.09 (13)	C19—C20—C22	112.93 (12)
C7—C3—H3	106.7	C21—C20—H20	107.1
C2—C3—H3	106.7	C19—C20—H20	107.1
C4—C3—H3	106.7	C22—C20—H20	107.1
C11—C4—C5	106.37 (13)	C20—C21—H21A	109.5
C11—C4—C3	109.29 (13)	C20—C21—H21B	109.5
C5—C4—C3	108.57 (12)	H21A—C21—H21B	109.5
C11—C4—C10	111.35 (12)	C20—C21—H21C	109.5
C5—C4—C10	112.31 (13)	H21A—C21—H21C	109.5
C3—C4—C10	108.87 (12)	H21B—C21—H21C	109.5
C6—C5—C4	115.09 (13)	C23—C22—C20	115.62 (12)
C6—C5—H5A	108.5	C23—C22—H22A	108.4
C4—C5—H5A	108.5	C20—C22—H22A	108.4
C6—C5—H5B	108.5	C23—C22—H22B	108.4
C4—C5—H5B	108.5	C20—C22—H22B	108.4
H5A—C5—H5B	107.5	H22A—C22—H22B	107.4
C1—C6—C5	108.26 (13)	C24—C23—C22	110.25 (12)
C1—C6—H6A	110.0	C24—C23—H23A	109.6
C5—C6—H6A	110.0	C22—C23—H23A	109.6
C1—C6—H6B	110.0	C24—C23—H23B	109.6
C5—C6—H6B	110.0	C22—C23—H23B	109.6
H6A—C6—H6B	108.4	H23A—C23—H23B	108.1
C8—C7—C3	114.92 (13)	O9—C24—C23	111.69 (12)
C8—C7—H7A	108.5	O9—C24—C25	90.95 (11)
C3—C7—H7A	108.5	C23—C24—C25	116.24 (13)
C8—C7—H7B	108.5	O9—C24—H24	112.1
C3—C7—H7B	108.5	C23—C24—H24	112.1
H7A—C7—H7B	107.5	C25—C24—H24	112.1
O3—C8—C9	105.53 (11)	C26—C25—C27	112.61 (14)
O3—C8—C7	110.99 (12)	C26—C25—C24	115.49 (14)
C9—C8—C7	111.99 (13)	C27—C25—C24	85.35 (12)
O3—C8—H8	109.4	C26—C25—H25	113.5
C9—C8—H8	109.4	C27—C25—H25	113.5
C7—C8—H8	109.4	C24—C25—H25	113.5
C8—C9—C15	112.19 (12)	O7—C26—C25	109.20 (14)
C8—C9—C10	109.44 (12)	O7—C26—H26A	109.8
C15—C9—C10	112.34 (12)	C25—C26—H26A	109.8
C8—C9—H9	107.5	O7—C26—H26B	109.8
C15—C9—H9	107.5	C25—C26—H26B	109.8
C10—C9—H9	107.5	H26A—C26—H26B	108.3
C12—C10—C9	113.74 (12)	O9—C27—C25	91.60 (12)
C12—C10—C4	113.44 (12)	O9—C27—H27A	113.4
C9—C10—C4	109.90 (12)	C25—C27—H27A	113.4
C12—C10—H10	106.4	O9—C27—H27B	113.4
C9—C10—H10	106.4	C25—C27—H27B	113.4
C4—C10—H10	106.4	H27A—C27—H27B	110.7
C4—C11—H11A	109.5	O2—C28—O1	123.57 (16)
C4—C11—H11B	109.5	O2—C28—C29	125.20 (16)
H11A—C11—H11B	109.5	O1—C28—C29	111.23 (14)
C4—C11—H11C	109.5	C28—C29—H29A	109.5
H11A—C11—H11C	109.5	C28—C29—H29B	109.5
H11B—C11—H11C	109.5	H29A—C29—H29B	109.5
C13—C12—C10	113.99 (12)	C28—C29—H29C	109.5
C13—C12—H12A	108.8	H29A—C29—H29C	109.5
C10—C12—H12A	108.8	H29B—C29—H29C	109.5
C13—C12—H12B	108.8	O4—C30—O3	124.10 (16)
C10—C12—H12B	108.8	O4—C30—C31	125.36 (15)
H12A—C12—H12B	107.7	O3—C30—C31	110.54 (14)
O5—C13—C14	107.30 (12)	C30—C31—H31A	109.5
O5—C13—C12	108.04 (12)	C30—C31—H31B	109.5
C14—C13—C12	111.55 (13)	H31A—C31—H31B	109.5
O5—C13—H13	110.0	C30—C31—H31C	109.5
C14—C13—H13	110.0	H31A—C31—H31C	109.5
C12—C13—H13	110.0	H31B—C31—H31C	109.5
C13—C14—C16	107.28 (12)	O6—C32—O5	123.22 (19)
C13—C14—C15	107.91 (11)	O6—C32—C33	125.41 (18)
C16—C14—C15	113.03 (13)	O5—C32—C33	111.36 (17)
C13—C14—C19	118.66 (12)	C32—C33—H33A	109.5
C16—C14—C19	110.07 (12)	C32—C33—H33B	109.5
C15—C14—C19	99.90 (11)	H33A—C33—H33B	109.5
C9—C15—C17	117.44 (12)	C32—C33—H33C	109.5
C9—C15—C14	113.63 (12)	H33A—C33—H33C	109.5
C17—C15—C14	103.95 (12)	H33B—C33—H33C	109.5
C9—C15—H15	107.1	O8—C34—O7	123.61 (17)
C17—C15—H15	107.1	O8—C34—C35	125.34 (17)
C14—C15—H15	107.1	O7—C34—C35	111.03 (15)
C14—C16—H16A	109.5	C34—C35—H35A	109.5
C14—C16—H16B	109.5	C34—C35—H35B	109.5
H16A—C16—H16B	109.5	H35A—C35—H35B	109.5
C14—C16—H16C	109.5	C34—C35—H35C	109.5
H16A—C16—H16C	109.5	H35A—C35—H35C	109.5
H16B—C16—H16C	109.5	H35B—C35—H35C	109.5
C15—C17—C18	104.04 (12)		
			
C28—O1—C1—C6	−159.24 (13)	C8—C9—C15—C17	−62.70 (17)
C28—O1—C1—C2	80.16 (16)	C10—C9—C15—C17	173.51 (12)
O1—C1—C2—C3	176.76 (12)	C8—C9—C15—C14	175.73 (12)
C6—C1—C2—C3	58.43 (17)	C10—C9—C15—C14	51.94 (16)
C1—C2—C3—C7	179.80 (12)	C13—C14—C15—C9	−59.54 (16)
C1—C2—C3—C4	−53.08 (17)	C16—C14—C15—C9	58.92 (16)
C7—C3—C4—C11	−69.76 (16)	C19—C14—C15—C9	175.84 (12)
C2—C3—C4—C11	164.02 (13)	C13—C14—C15—C17	171.63 (12)
C7—C3—C4—C5	174.61 (12)	C16—C14—C15—C17	−69.90 (15)
C2—C3—C4—C5	48.39 (16)	C19—C14—C15—C17	47.02 (14)
C7—C3—C4—C10	52.06 (16)	C9—C15—C17—C18	−156.59 (13)
C2—C3—C4—C10	−74.16 (15)	C14—C15—C17—C18	−30.13 (15)
C11—C4—C5—C6	−170.10 (13)	C15—C17—C18—C19	1.41 (16)
C3—C4—C5—C6	−52.59 (17)	C17—C18—C19—C20	154.78 (12)
C10—C4—C5—C6	67.85 (17)	C17—C18—C19—C14	27.19 (14)
O1—C1—C6—C5	−179.27 (12)	C13—C14—C19—C20	73.75 (17)
C2—C1—C6—C5	−59.50 (17)	C16—C14—C19—C20	−50.32 (17)
C4—C5—C6—C1	58.59 (17)	C15—C14—C19—C20	−169.43 (12)
C2—C3—C7—C8	80.19 (16)	C13—C14—C19—C18	−161.62 (12)
C4—C3—C7—C8	−47.32 (18)	C16—C14—C19—C18	74.31 (14)
C30—O3—C8—C9	151.13 (13)	C15—C14—C19—C18	−44.81 (13)
C30—O3—C8—C7	−87.34 (16)	C18—C19—C20—C21	171.99 (13)
C3—C7—C8—O3	−68.55 (16)	C14—C19—C20—C21	−69.42 (17)
C3—C7—C8—C9	49.11 (17)	C18—C19—C20—C22	46.78 (17)
O3—C8—C9—C15	−60.65 (15)	C14—C19—C20—C22	165.36 (13)
C7—C8—C9—C15	178.47 (12)	C21—C20—C22—C23	−61.34 (17)
O3—C8—C9—C10	64.75 (15)	C19—C20—C22—C23	64.81 (18)
C7—C8—C9—C10	−56.13 (16)	C20—C22—C23—C24	170.42 (13)
C8—C9—C10—C12	−168.46 (12)	C27—O9—C24—C23	110.70 (14)
C15—C9—C10—C12	−43.15 (17)	C27—O9—C24—C25	−8.14 (12)
C8—C9—C10—C4	63.12 (15)	C22—C23—C24—O9	179.66 (12)
C15—C9—C10—C4	−171.56 (12)	C22—C23—C24—C25	−77.87 (17)
C11—C4—C10—C12	−68.69 (17)	O9—C24—C25—C26	−105.13 (15)
C5—C4—C10—C12	50.48 (17)	C23—C24—C25—C26	140.01 (15)
C3—C4—C10—C12	170.75 (12)	O9—C24—C25—C27	7.74 (11)
C11—C4—C10—C9	59.89 (17)	C23—C24—C25—C27	−107.12 (15)
C5—C4—C10—C9	179.06 (12)	C34—O7—C26—C25	−125.74 (16)
C3—C4—C10—C9	−60.67 (15)	C27—C25—C26—O7	−158.84 (15)
C9—C10—C12—C13	44.55 (18)	C24—C25—C26—O7	−63.0 (2)
C4—C10—C12—C13	171.13 (13)	C24—O9—C27—C25	8.17 (12)
C32—O5—C13—C14	−152.07 (13)	C26—C25—C27—O9	107.91 (15)
C32—O5—C13—C12	87.54 (15)	C24—C25—C27—O9	−7.81 (11)
C10—C12—C13—O5	64.29 (16)	C1—O1—C28—O2	4.1 (2)
C10—C12—C13—C14	−53.40 (17)	C1—O1—C28—C29	−175.51 (13)
O5—C13—C14—C16	178.76 (11)	C8—O3—C30—O4	1.5 (2)
C12—C13—C14—C16	−63.10 (15)	C8—O3—C30—C31	−179.50 (12)
O5—C13—C14—C15	−59.16 (15)	C13—O5—C32—O6	2.6 (2)
C12—C13—C14—C15	58.98 (16)	C13—O5—C32—C33	−176.65 (13)
O5—C13—C14—C19	53.33 (16)	C26—O7—C34—O8	−0.3 (3)
C12—C13—C14—C19	171.47 (12)	C26—O7—C34—C35	178.09 (16)
